# A Nomogram Based on the Log Odds of Positive Lymph Nodes Predicts the Prognosis of Patients With Distal Cholangiocarcinoma After Surgery

**DOI:** 10.3389/fsurg.2021.757552

**Published:** 2021-10-26

**Authors:** Rui Li, Zhenhua Lu, Zhen Sun, Xiaolei Shi, Zhe Li, Weiwei Shao, Yangyang Zheng, Jinghai Song

**Affiliations:** ^1^Department of General Surgery, Department of Hepato-Bilio-Pancreatic Surgery, National Center of Gerontology, Institute of Geriatric Medicine, Beijing Hospital, Peking Union Medical College, Chinese Academy of Medical Sciences, Beijing, China; ^2^9th Department, Plastic Surgery Hospital, Peking Union Medical College, Chinese Academy of Medical Sciences, Beijing, China; ^3^The Key Laboratory of Geriatrics, National Center of Gerontology, National Health Commission, Beijing Institute of Geriatrics, Beijing Hospital, Beijing, China; ^4^Institute of Geriatric Medicine, Chinese Academy of Medical Sciences, Beijing, China; ^5^Division of Colorectal Surgery, Department of General Surgery, Peking Union Medical College Hospital, Chinese Academy of Medical Sciences, Beijing, China

**Keywords:** distal cholangiocarcinoma, log odds of positive lymph nodes, lymph node ratio, nomogram, prognosis

## Abstract

**Background:** Lymph node (LN) metastasis is considered one of the most important risk factors affecting the prognosis of distal cholangiocarcinoma (DCC). This study aimed to demonstrate the superiority of log odds of positive lymph nodes (LODDS) compared with other LN stages, and to establish a novel prognostic nomogram to predict the cancer-specific survival (CSS) of DCC.

**Methods:** From the Surveillance, Epidemiology and End Results (SEER) database, the data of 676 patients after DCC radical operation were screened, and patients were randomly divided into training (*n* = 474) and validation sets (*n* = 474). The prognostic evaluation performance of the LODDS and American Joint Commission on Cancer (AJCC) N stage and lymph node ratio (LNR) were compared using the Akaike information criteria, receiver operating characteristic area under the curve (AUC), and C-index. Multivariate Cox analysis was used to screen independent risk factors, and a LODDS-based nomogram prognostic staging model was established. The nomogram's precision was verified by C-index, calibration curves, and AUC, and the results were compared with those of the AJCC TNM staging system.

**Results:**Compared with the other two stages of LN metastasis, LODDS was most effective in predicting CSS in patients with DCC. Multivariate analysis proved that LODDS, histologic grade, SEER historic stage, and tumor size were independent risk factors for DCC. The C-index of the nomogram, based on the above factors, in the validation set was 0.663. The 1-, 3-, and 5-y AUCs were 0.735, 0.679, and 0.745, respectively. Its good performance was also verified by calibration curves. In addition, the C-index and Kaplan-Meier analysis showed that the nomogram performed better than the AJCC TNM staging system.

**Conclusion:**For postoperative patients with DCC, the LODDS stage yielded better prognostic efficiency than the AJCC N and LNR stages. Compared with the AJCC TNM staging system, the nomogram, based on the LODDS, demonstrated superior performance.

## Introduction

Cholangiocarcinoma accounts for ~15% of all hepatobiliary tumors (1). According to the tumor location, cholangiocarcinoma is divided into three categories: intrahepatic cholangiocarcinoma, perihilar cholangiocarcinoma, and distal cholangiocarcinoma (DCC). Among them, distal cholangiocarcinoma accounts for ~30% of cholangiocarcinomas. Radical resection is the most important method for treatment of DCC, but even the 5-y survival rate after surgery is still unsatisfactory, ranging from 16 to 39.5% (2–6). Lymph node (LN) metastasis is an independent prognostic factor for DCC, and some studies have shown that it is the single most important factor (7–10). Therefore, accurate and efficient evaluation indicators for LN metastasis are necessary to provide patients with individualized treatment and improve the prognosis of patients with DCC.

At present, the most common predictive indicator for LN metastasis is the N stage, as proposed by the American Joint Committee on Cancer (AJCC). In the 8th edition of the latest AJCC TNM staging system, the rules of N staging were significantly changed. Instead of simply dividing them into non-regional LN metastasis (N0) and regional LN metastasis (N1), the staging system is divided into N0 (positive lymph node number [PLNN] = 0), N1 (1 ≤ PLNN ≤ 3), and N2 (PLNN≥4) (11). However, staging regional LN metastasis based on PLNN and ignoring the actual number of examined lymph nodes (ELNN) during surgery may lead to bias. Therefore, some scholars have proposed that lymph node ratio (LNR) and log odds of positive lymph nodes (LODDS) are better staging criteria for LN metastasis than N stage in a variety of tumors, such as thyroid, colorectal, and pancreatic cancer (12–15). LNR is defined as the ratio of PLNN to ELNN; LODDS is defined as the log of the quotient of the PLNN and the number of negative LNs (14–16).

To the best of our knowledge, no study has compared the prognostic value of N stage, LNR, and LODDS in patients with DCC. This study aimed to evaluate the accuracy of LODDS in predicting LN metastasis based on the SEER database. In addition, by incorporating LODDS and other independent risk factors, a novel nomogram was established to predict the prognosis of patients with DCC in the training set. Finally, the performance of the nomogram in the validation set was tested and compared with the AJCC TNM staging system.

## Materials and Methods

### Data Collection

The data included in this study were extracted from the SEER 18 registries research database, and all enrolled patients were diagnosed with distal cholangiocarcinoma who received radical surgery between 2004 and 2015. The database collects cancer diagnosis, treatment, and survival data for ~30% of the United States population. Inclusion criteria were as follows: (1) patients classified as having “Bile Ducts Distal” according to “CS schema v0204+”; (2) patients older than 18 years at diagnosis; (3) patients with complete LN biopsy records; and (4) patients with survival time more than 1 mo. The exclusion criteria were as follows: (1) an unconfirmed diagnosis by histopathology; (2) an incomplete clinicopathological data; and (3) patients who had died of causes other than DCC, or an unknown cause. Ultimately, 676 patients with DCCs were enrolled in this study. The patients were randomly divided into a training set (*n* = 474) and a validation set (*n* = 202) at a ratio of 7:3. This study has been registered in Beijing Hospital, and the Beijing Hospital Medical Ethics Committee approved the study.

The following data were obtained: sex, age, race, year of diagnosis, histologic grade, SEER historic stage, histology, tumor size, AJCC TNM stage, T/N/M stage, ELNN, PLNN, LNR, and LODDS. The calculation formulas of LNR and LODDS are as follows: LNR = PLNN/ELNN; LODDS= log[(PLNN+0.05)/(ELNN-PLNN+0.05)]. Cancer-specific survival (CSS), defined as the date of diagnosis to the date of death from DCCs, was set as the end point of this study.

### Optimal Cut-Off Points of the Variables

The best cut-off values for tumor size, LNR, and LODDS were calculated using the X-tile 3.6.1 program based on the principles of maximum chi-square value and minimum *p*-value. The best cut-off points for tumor size were 14 and 33 mm. The best cut-off points of LNR were 0.11 and 0.33; hence, LNR was divided into three groups, LNR1 (LNR ≤ 0.11), LNR2 (0.12 < LNR ≤ 0.33), and LNR3 (>0.33). As the best cut-off points of LODDS were −2.00 and −0.29, LODDS was divided into three groups: LODDS1 (LODDS ≤ -2.00), LODDS2 (−2.00 < LODDS ≤ 0.29), and LODDS3 (LODDS>0.29).

### Statistical Analysis

Kaplan-Meier curves and log-rank tests were used to evaluate the effectiveness of the N stage, LNR, and LODDS for prognostic stratification. The C-index, Akaike information criterion (AIC), and receiver operating characteristic (ROC) area under the ROC curve (AUC) were applied to compare the predictive performance of N stage, LNR, and LODDS. Univariate and multivariate Cox analyses were used to identify independent risk factors for DCC. Based on the results of multivariate analysis, a nomogram was constructed to predict the 1-, 3-, and 5-y CSS of patients with DCC. Additionally, the AUC, calibration curves, AIC, and C-index were applied to verify the predictive performance of the nomogram and compare it with the AJCC TNM staging system. Furthermore, according to the nomogram, the risk scores of all patients in the training group were calculated, and X-tile 3.6.1 was used for risk stratification, which was divided into three stages: stage I, stage II and stage III. Kaplan-Meier analysis and log-rank test were used to compare the differences in survival at each stage and compared with the AJCC TNM staging system.

Statistical analyses were carried out using R software 4.0.3 (R Foundation, Vienna, Austria) and SPSS 24.0 (SPSS, Chicago, IL). Statistical significance was defined as a two-tailed *p* < 0.05.

## Results

### Clinicopathological Characteristics

From 2004 to 2015, a total of 676 patients with radically resected DCCs were included in this study. They were randomly divided into a training set (*n* = 474) and validation set (*n* = 202) at a ratio of 7:3. Table 1 summarizes the clinical and pathological characteristics of patients with DCC. The median follow-up time was 53 months (range: 1–162 months), and the 1, 3, and 5-y CSS rates were 82.8, 47.9, and 36.2%, respectively. The entire cohort (*n* = 676) included 429 (63.5%) men and 497 (73.5%) white patients. A total of 524 (77.5%) patients were aged >60 years at the time of diagnosis; 658 (97.3%) were diagnosed between 2010 and 2015 and 626 (92.6%) patients were AJCC TNM stage I-II. The median tumor size was 21 mm (range: 1–80 mm).

**Table 1:** Demographics and clinicopathologic characteristics of patients.

**Variables**	**Total number, *n* (%)**	**Training cohort, *n* (%)**	**Validation cohort, *n* (%)**
	**(*n =* 676)**	**(*n =* 474)**	**(*n =* 202)**
**Sex**			
Female	247 (36.5)	179 (37.8)	68 (33.7)
Male	429 (63.5)	295 (62.2)	134 (66.3)
**Age (years)**			
<60	152 (22.5)	110 (23.2)	42 (20.8)
60–80	445 (65.8)	303 (63.9)	142 (70.3)
≥80	79 (11.7)	61 (12.9)	18 (8.9)
**Race**			
White	497 (73.5)	349 (73.6)	148 (73.3)
Black	53 (7.8)	41 (8.6)	12 (5.9)
Others	126 (18.6)	84 (17.7)	42 (20.8)
**Year of diagnosis (years)**			
2004–2009	18 (2.7)	15 (3.2)	3 (1.5)
2010–2015	658 (97.3)	459 (96.8)	199 (98.5)
**Grade**			
Well	84 (12.4)	68 (14.3)	16 (7.9)
Moderate	333 (49.3)	234 (49.4)	99 (49.0)
Poor	252 (37.3)	169 (35.7)	83 (41.1)
Undifferentiated	7 (1.0)	3 (0.6)	4 (2.0)
**Historic stage**			
Localized	61 (9.0)	38 (8.0)	23 (11.4)
Regional	583 (86.2)	407 (85.9)	176 (87.1)
Distant	32 (4.7)	29 (6.1)	3 (1.5)
**Histology**			
Adenocarcinoma	407 (60.2)	286 (60.3)	121 (59.9)
Others	51 (7.5)	34 (7.2)	17 (8.4)
Unknown	218 (32.2)	154 (32.5)	64 (31.7)
**Tumor size (mm)**			
1–14	133 (19.7)	93 (19.6)	40 (19.8)
15–33	426 (63.0)	303 (63.9)	123 (60.9)
34–80	117 (17.3)	78 (16.5)	39 (19.3)
**AJCC TNM stage**			
I	154 (22.8)	114 (24.1)	40 (19.8)
II	472 (69.8)	323 (68.1)	149 (73.8)
III	25 (3.7)	15 (3.2)	10 (5.0)
IV	25 (3.7)	22 (4.6)	3 (1.5)
**T stage**			
T1	74 (10.9)	48 (10.1)	26 (12.9)
T2	146 (21.6)	117 (24.7)	29 (14.4)
T3	430 (63.6)	293 (61.8)	137 (67.8)
T4	26 (3.8)	16 (3.4)	10 (5.0)
**N stage**			
N0	330 (48.8)	226 (47.7)	104 (51.5)
N1	247 (36.5)	177 (37.3)	70 (34.7)
N2	99 (14.6)	71 (15.0)	28 (13.9)
**M stage**			
M0	651 (96.3)	452 (95.4)	199 (98.5)
M1	25 (3.7)	22 (4.6)	3 (1.5)
**LNR**			
LNR1	452 (66.9)	310 (65.4)	142 (70.3)
LNR2	145 (21.4)	111 (23.4)	34 (16.8)
LNR3	79 (11.7)	53 (11.2)	26 (12.9)
**LODDS**			
LODDS1	244 (36.1)	166 (35.0)	78 (38.6)
LODDS2	353 (52.2)	255 (53.8)	98 (48.5)
LODDS3	79 (11.7)	53 (11.2)	26 (32.9)

*LNR, positive lymph node ratio; LODDS, log odds of positive lymph nodes*.

The median ELNN was 14 (range: 1–63), and 288 (42.6%) patients had ELNN <12, which means that nearly half of the patients had insufficient regional LNs retrieved (AJCC recommended that at least 12 LNs be retrieved); thus, it was difficult to obtain an accurate N stage. At the same time, more than half of the patients (346, 51.1%) had regional LN metastasis. The median LNR was 0.03 (range: 0–1). The median LODDS was −1.32 (range: −3 to 2).

### Prognostic Factors for CSS in DCC Patients

**Figure 1:**
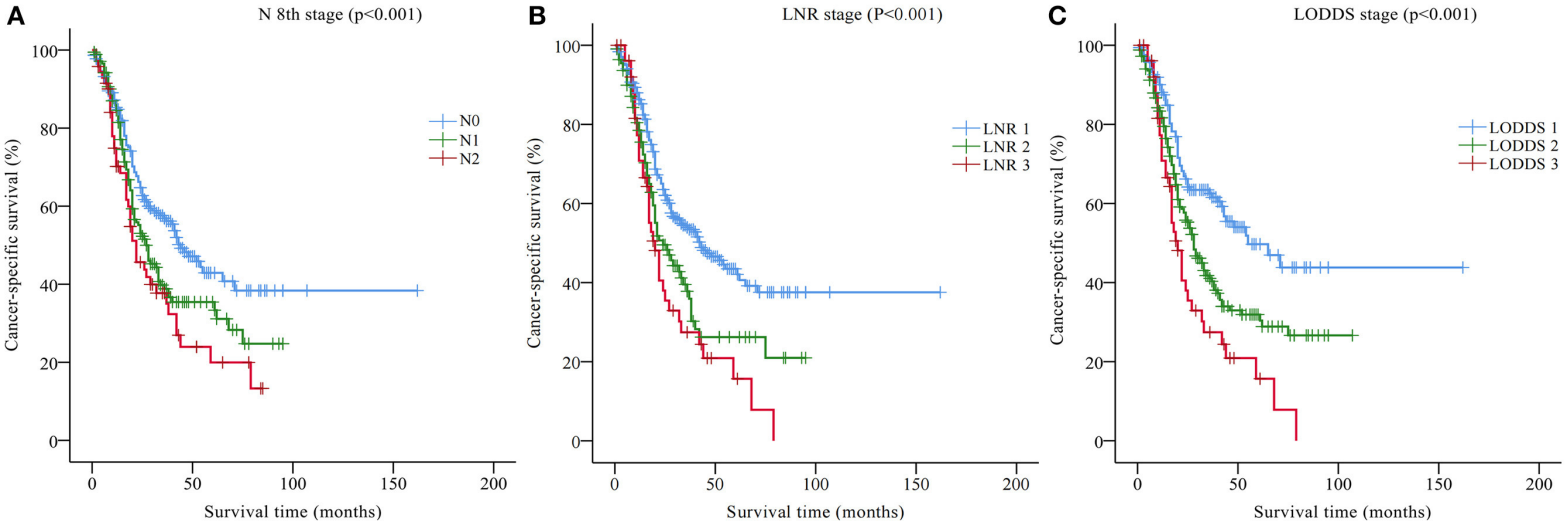
Kaplan-Meier CSS curves stratified by **(A)** N 8th stage, **(B)** the LNR stage, and **(C)** the LODDS stage. CSS, cancer-specific survival; LNR, lymph node ratio; LODDS, log odds of positive lymph nodes.

Univariate analysis based on the training set showed that nine variables were closely related to the CSS of DCC patients: tumor grade, SEER historic stage, tumor size, AJCC TNM stage, T stage, N stage, M stage, LNR, and LODDS (*p* < 0.05, Table 2). In addition, Kaplan-Meier survival analysis and log-rank test revealed that the prognosis of patients with DCC could be significantly stratified by N stage, LNR, or LODDS (*p* < 0.001, Figure 1).

**Table 2:** Uni- and multivariate analysis of prognostic factors associated with cancer-specific survival for patients in the training cohort with distal cholangiocarcinoma.

**Variables**	**Univariate analysis**		**Multivariate analysis**	
	**HR (95% CI)**	***P*-value**	**HR (95% CI)**	***P*-value**
**Sex**
Female	Reference			
Male	1.140 (0.880–1.476)	0.321		
**Age (years)**				
<60	Reference			
60–80	0.950 (0.709–1.274)	0.733		
≥80	0.922 (0.579–1.467)	0.731		
**Race**				
White	Reference			
Black	0.764 (0.487–1.200)	0.243		
Others	0.836 (0.592–1.181)	0.310		
**Year of diagnosis (years)**				
2004–2009	Reference			
2010–2015	1.155 (0.545–2.451)	0.707		
**Grade**				
Well	Reference		Reference	
Moderate	1.454 (0.968–2.185)	0.071	1.168 (0.767–1.780)	0.469
Poor	1.907 (1.258–2.889)	0.002	1.554 (1.013–2.384)	0.043
Undifferentiated	6.449 (1.519–27.377)	0.012	3.879 (0.891–16.885)	0.071
**Historic stage**				
Localized	Reference		Reference	
Regional	2.597 (1.377–4.899)	0.003	1.540 (0.509–4.659)	0.445
Distant	5.144 (2.432–10.882)	<0.001	4.746 (1.125–20.014)	0.034
**Histology**				
Adenocarcinoma	Reference			
Others	1.237 (0.782–1.956)	0.363		
Unknown	1.061 (0.810–1.390)	0.668		
**Tumor size (mm)**				
1–14	Reference		Reference	
15–33	2.031 (1.385–2.980)	<0.001	1.786 (1.204–2.650)	0.004
34–80	2.541 (1.613–4.001)	<0.001	2.123 (1.324–3.403)	0.002
**AJCC TNM stage**				
I	Reference			
II	1.772 (1.273–2.467)	0.001		
III	2.183 (1.098–4.341)	0.026		
IV	2.889 (1.648–5.065)	<0.001		
**T stage**				
T1	Reference		Reference	
T2	1.505 (0.847–2.674)	0.164	0.802 (0.315–2.044)	0.644
T3	2.435 (1.435–4.134)	0.001	1.180 (0.478–2.915)	0.720
T4	2.872 (1.318–6.255)	0.008	1.232 (0.420–3.614)	0.704
**N stage**				
N0	Reference			
N1	1.435 (1.088–1.892)	0.011		
N2	1.838 (1.295–2.610)	0.001		
**M stage**				
M0	Reference		Reference	
M1	1.853 (1.131–3.035)	0.014	0.539 (0.195–1.487)	0.232
**LNR**				
LNR1	Reference			
LNR2	1.592 (1.187–2.134)	0.002		
LNR3	2.076 (1.445–2.983)	<0.001		
**LODDS**				
LODDS1	Reference		Reference	
LODDS2	1.689 (1.264–2.257)	<0.001	1.476 (1.095–1.988)	0.011
LODDS3	2.539 (1.699–3.796)	<0.001	1.834 (1.198–2.808)	0.005

*HR, hazard ratio; CI, confidence interval; LNR, positive lymph node ratio; LODDS, log odds of positive lymph nodes*.

Table 3 compares the prognostic prediction performance of the N stage, LNR, and LODDS according to the training set. The C-indices of the N stage, LNR, and LODDS were 0.560, 0.564, and 0.573, respectively. The AICs of the N stage, LNR, and LODDS were 2,733, 2,727, and 2,722, respectively. The AUCs of the N stage, LNR, and LODDS predictive of 1-y CSS were 0.571, 0.588, and 0.589, respectively. The AUCs of the N stage, LNR, and LODDS predictive of 3-y CSS were 0.597, 0.602, and 0.630, respectively. The AUCs of the N stage, LNR, and LODDS predictive of 5-y CSS were 0.609, 0.588, and 0.629, respectively. In short, the C-index and AUC of LODDS were the highest among the three, whereas AIC was the lowest, indicating that the predictive performance of LODDS on CSS was better than that of N stage and LNR in DCC patients. Therefore, LODDS was included in the multivariate analysis, and the results showed that grade, SEER historic stage, tumor size, and LODDS were independent risk factors that affected CSS in patients with DCC (*p* < 0.05, Table 2).

**Table 3:** Prognostic efficiency of different lymph node staging systems.

**Systems**	**C-index**	**AIC**	**AUC**		
			**1-year CSS**	**3-year CSS**	**5-year CSS**
N stage	0.560	2,733	0.571	0.597	0.609
LNR stage	0.564	2,727	0.588	0.602	0.588
LODDS stage	0.573	2,722	0.589	0.630	0.629

*C-index, concordance index; AIC, Akaike information criterion; AUC, area under the receiver operating characteristic curve; LNR, positive lymph node ratio; LODDS, log odds of positive lymph nodes*.

### Construction and Validation of the Prognostic Nomogram for CSS

**Figure 2:**
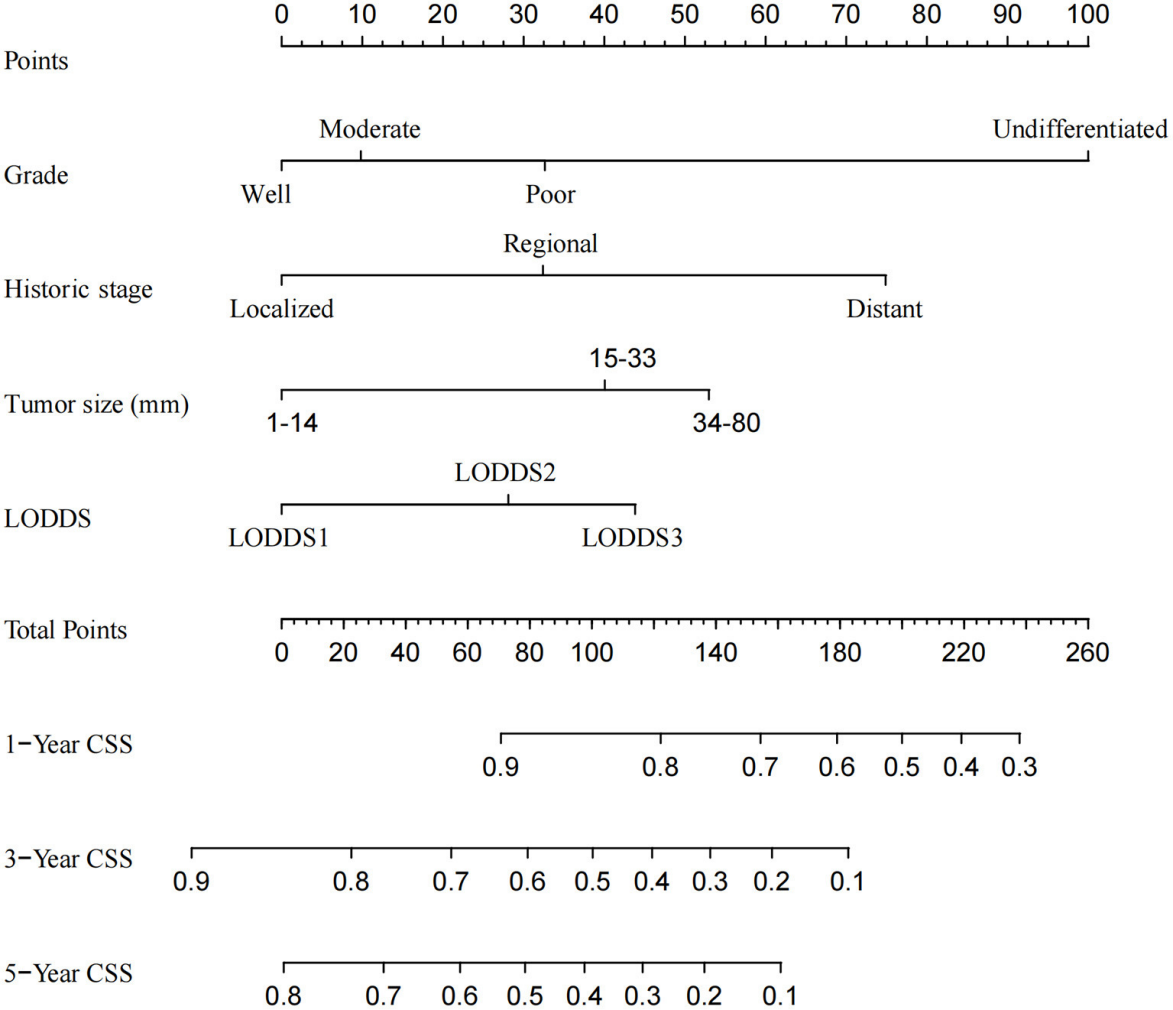
Nomogram for predicting the 1-, 3-, and 5-year cancer-specific survival in patients with distal cholangiocarcinoma.

**Figure 3:**
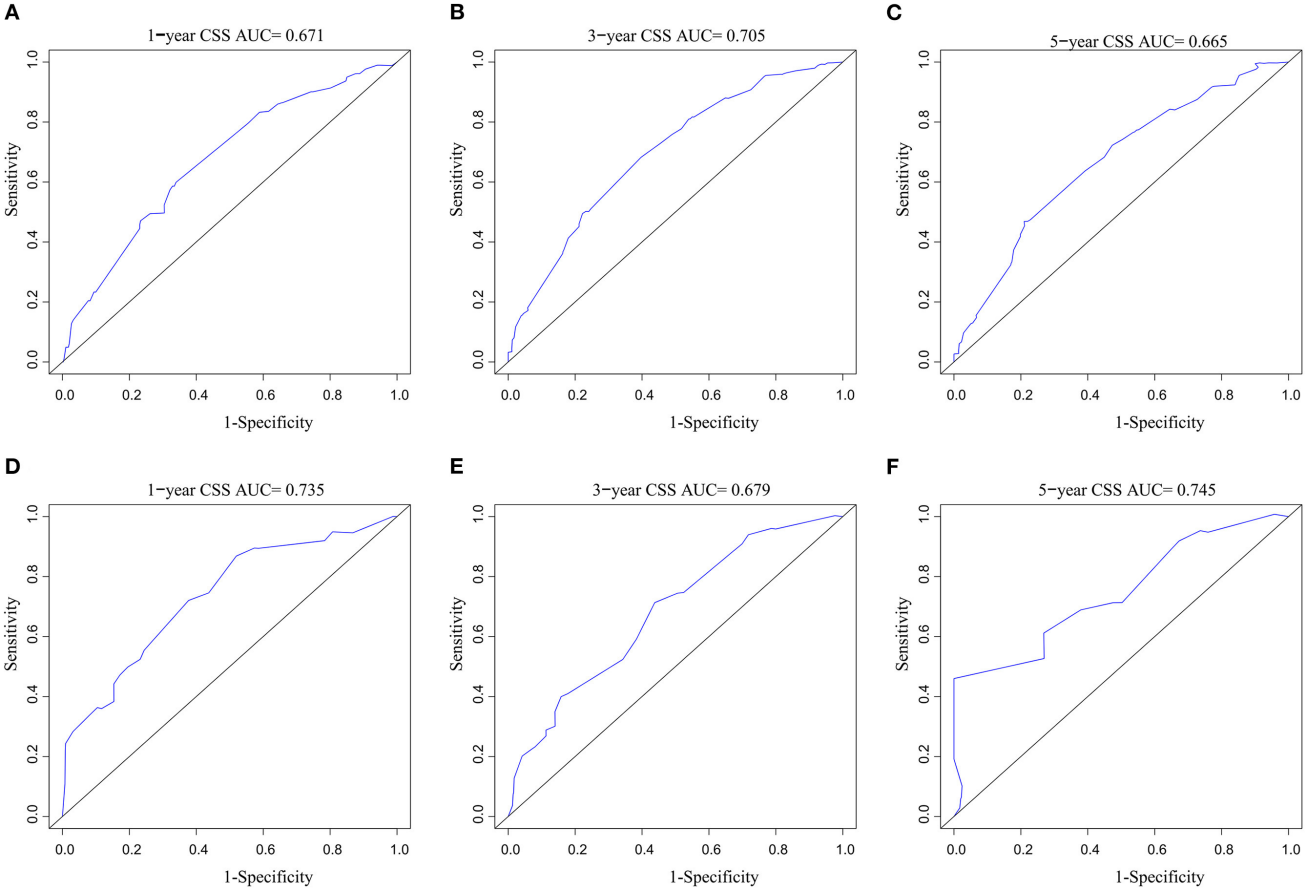
AUC value of ROC predicting: **(A)** 1-year CSS of the nomogram in the training set; **(B)** 3-year CSS of the nomogram in the training set; **(C)** 5-year CSS of the nomogram in the training set; **(D)** 1-year CSS of the nomogram in the validation set; **(E)** 3-year CSS of the nomogram in the validation set; **(F)** 5-year CSS of the nomogram in the validation set. AUC, area under the curve; ROC, receiver operating characteristic; CSS, cancer-specific survival.

**Figure 4:**
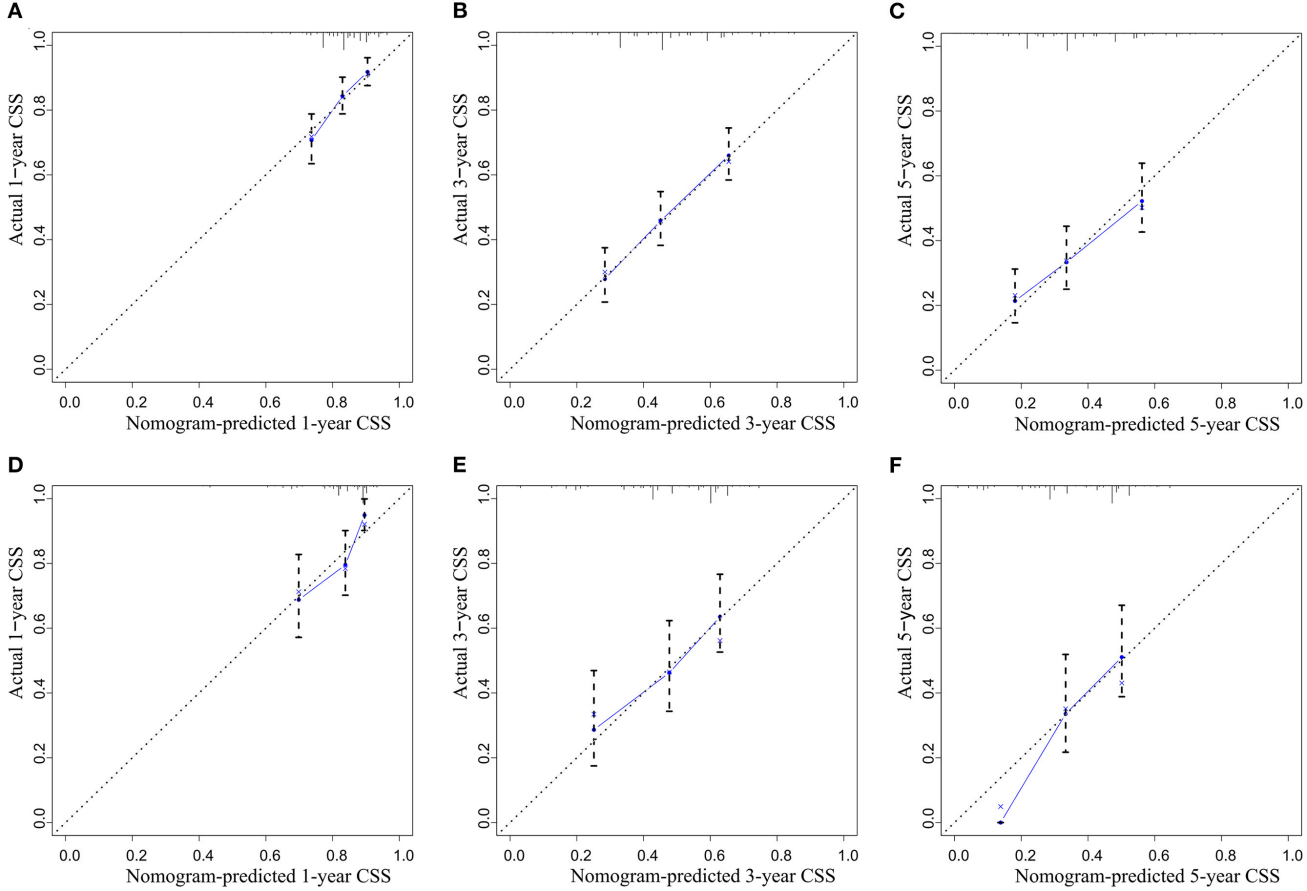
The calibration curve for predicting CSS: **(A)** at 1 year in the training set; **(B)** at 3 years in the training set; **(C)** at 5 years in the training set; **(D)** at 1 year in the validation set; **(E)** at 3 years in the validation set; **(F)** at 5 years in the validation set.

A CSS prognostic nomogram was constructed (Figure 2) according to the four independent risk factors, which includes grade, SEER historic stage, tumor size, and LODDS. In the training set, the AUC values of the nomogram for predicting 1-, 3-, and 5- y CSS were 0.671, 0.705, and 0.665, respectively (Figures 3A–C); similar results were observed in the validation set, and the AUC values of the nomogram for predicting 1-, 3-, and 5-y CSS were 0.735, 0.679, and 0.745, respectively (Figures 3D–F). The calibration curves showed an optimal agreement between the nomogram-predicted and actual observed 1-, 3-, and 5-y CSS in both the training set (Figures 4A–C) and the validation set (Figures 4D–F).

Comparing the performance of the nomogram and AJCC TNM stage, in the training set, the C-index of the nomogram for CSS prediction was 0.639 (95% CI, 0.728–0.798), which was significantly higher than AJCC TNM 0.577 (95% CI, 0.685–0.751), and the AIC of the nomogram is 2702, which is lower than AJCC TNM 2729; the results in the validation set were similar, and the C-index of the nomogram was 0.663 (95% CI, 0.728–0.838), which was significantly higher than AJCC TNM 0.516 (95% CI, 0.694–0.796), and the AIC of the nomogram is 930, which is lower than AJCC TNM 944. In short, both indicators indicate that the nomogram is a more accurate prognostic model than the AJCC TNM stage.

### Risk Stratification Based on the Nomogram

**Figure 5:**
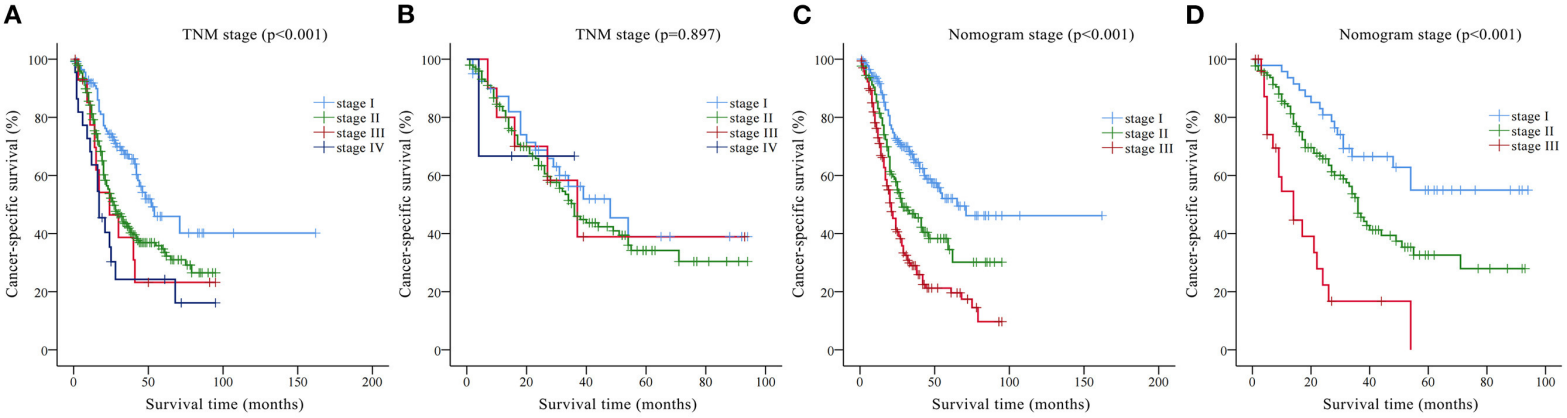
Kaplan-Meier CSS curves categorized by different staging systems: **(A)** American Joint Committee on Cancer (AJCC) TNM staging system in the training set; **(B)** AJCC TNM staging system in the validation set; **(C)** nomogram staging system in the training set; **(D)** nomogram staging system in the validation set.

According to the nomogram, all patients in the training set were risk-scored, and were divided into three stages according to risk stratification: stage I (0–95.2), stage II (95.2–116.3) and stage III (>116.3). Kaplan-Meier curves were drawn for the nomogram staging system and AJCC TNM staging system according to the training and validation sets, respectively (Figure 5). In the training set, although the Kaplan-Meier curves of the two staging systems both showed prognostic stratification (log-rank *p* < 0.001), some significant overlaps of survival curves were observed in the AJCC TNM staging system (Figures 5A,B). In the validation set, the AJCC TNM staging system did not even have an obvious prognostic stratification (log-rank *p* = 0.897, Figure 5C), whereas the prognostic stratification of the nomogram staging system was still very clear (log-rank *p* < 0.001, Figure 5D).

## Discussion

Regional LN metastasis is considered to be a key factor in evaluating the prognosis of patients after DCC (7–10, 17), but the effectiveness of the AJCC 8th edition N stage, as the latest and most commonly used stage, remains controversial (7, 17–19). Compared with AJCC 7th, AJCC 8th edition further subdivided N stage into N0 (PLNN = 0), N1 (PLNN = 1–3) and N2 (PLNN≥4) according to PLNN (11). However, the actual number of retrieved LNs during surgery is often insufficient (AJCC recommends that at least 12 LNs be retrieved). For example, in this study, enough LNs could not be retrieved for 42.6% of patients, which would seriously interfere with the accuracy of the AJCC 8th edition N stage (20, 21). Therefore, an LNR stage has been proposed. Chin et al. reported that node positivity in itself does not provide optimal prognostic granularity in patients with DCC. LNR represents a hybrid parameter that accounts for both PLNN to ELNN is significantly associated with OS in DCC patients undergoing curative therapy (7, 18, 22). However, the LNR also has shortcomings. First, for patients for whom positive LNs could not retrieve (LNR = 0 and AJCC N0), the stage is indistinguishable (23). Second, the number of LN metastases in some patients is not large, and only positive LNs were retrieved during the operation, but negative LNs were not retrieved. At this time, PLNN is equal to ELNN (LNR = 1), such that patients with few positive LNs are classified as having an LNR stage that is too high, as confirmed by many studies (24, 25). Therefore, some scholars have constructed the LODDS stage. The LODDS stage considers both PLNN and negative LNs, which means that LODDS can be used in situations where LNR is difficult to stratify (23–25). However, no research has explored the prognostic significance of the LODDS stage in patients with DCC and its staging accuracy.

In this study, univariate and multivariate analyses showed that LODDS was significantly related to CSS in patients with DCC. The X-tile software was used to stratify the LODDS risk score based on the principle of the largest chi-square value and the smallest *p*-value to obtain a novel LODDS stage. In addition, through Kaplan-Meier survival analysis, the LODDS stage was not only proved to be closely related to the prognosis of DCC, but was also an important factor in DCC risk stratification. The higher the LODDS stage, the lower was the CSS. According to the evaluation criteria composed of C-index, AIC, and AUC, we also found that the prognostic prediction performance of the LODDS stage was better than that of the traditional N stage and LNR stage. This conclusion is consistent with that of many previous studies, but the specific staging method for LODDS is still inconclusive. For example, the LODDS stage of medullary thyroid cancer (13) is LODDS1 ( ≤ −0.9), LODDS2 (−0.9~-0.1) and LODDS3 (>-0.1); for gastric cancer (26) is LODDS1 ( ≤ -3), LODDS2 (−3~-1), LODDS3 (−1~3) and LODDS4 (> 3); perihilar cholangiocarcinoma (27) is LODDS1 (−3.11~-1.32), LODDS2 (−1.32~-0.39) and LODDS3 (−0.39~2.45); gallbladder cancer (28) is LODDS1 ( ≤ -1.5), LODDS2 (−1.5~0), LODDS3 (0~1.5) and LODDS3 (≥ 1.5). In this study, the LODDS stages of DCC were LODDS1 ( ≤ -2.00), LODDS2 (−2.00~0.29), and LODDS3 (>0.29). The stages of LODDS are not the same in different cancers, which may be due to the different aggressiveness of different cancers and the different patterns of LN metastasis in different cancers.

Multivariate analysis also indicated that in addition to LODDS stage, tumor grade, SEER historic stage, and tumor size were also independent risk factors for DCC patients. The prognostic value of these factors has been verified in a variety of tumors, including DCCs (4, 29–31). Whether age is an independent risk factor for DCC remains controversial. You et al. (19) reported that age was significantly related to the prognosis of DCC, but Chen et al. (30), Wu et al. (31), and the results of this study reached the opposite conclusion. This may be due to the limitations of the different studies. It is vital to look forward to future prospective, multicenter, large-sample studies to further explore this issue.

A prognostic nomogram, a visualization tool for assessing the survival probability of DCCs, was established based on the large-sample SEER database, where four independent risk factors (LODDS stage, grade, SEER historic stage, and tumor size) were included. Then, using the C-index, calibration curve, and ROC curve, it was confirmed that the nomogram had better CSS prediction performance for patients with DCC than the traditional AJCC TNM staging system. Finally, the training set patients were staged according to the nomogram, and Kaplan-Meier analysis and log-rank test proved that the staging accuracy of the nomogram model was significantly better than that of the AJCC TNM staging system, which helps guide clinicians to develop individualized treatment plans to improve the prognosis of patients with DCC.

To our knowledge, this study has proved for the first time that the LODDS stage is a more reliable prognostic staging indicator than the AJCC 8th edition N stage and LNR stage. At the same time, our novel LODDS-based nomogram model has been proved to be more accurate and intuitive than the traditional AJCC TNM staging system. However, this study has some limitations. First, although the sample size of this study was large, it was a retrospective study, and the conclusions need to be verified by prospective studies. Second, some potential prognostic factors of DCCs are not included in the SEER database, such as jaundice, resection margin status, and adjuvant therapy. Finally, because the latest AJCC 8th edition T staging standard has been redefined, the specific value of tumor invasion depth has replaced the traditional anatomical level of the bile duct, and the SEER database is difficult to update for this change in the short term; hence, the AJCC 7th edition T stage was still used in this study.

## Conclusions

For postoperative patients with DCC, LODDS is an independent risk factor for CSS, and its prognostic value is higher than that of the traditional AJCC 8th edition N stage and LNR stage. In addition, based on the SEER database, a prognostic nomogram model was established and verified using LODDS, grade, SEER historic stage, and tumor size. This visualization tool for assessing the survival probability of DCC will assist clinicians in providing patients with more individualized and precise treatment and follow-up.

## Data Availability Statement

The data analyzed in this study were obtained from the Surveillance, Epidemiology, and End Results (SEER) database. The following licenses/restrictions apply: requests to access these datasets must first be approved by SEER. Requests to access these datasets should be directed at https://seer.cancer.gov/data/access.html.

## Author Contributions

RL and ZLu were involved in the final data analysis and manuscript writing. ZS, XS, ZLi, WS, and YZ were assisted in the data collection and analysis. JS was involved in study design and responsible for the entire research project. All authors have agreed on the journal to which the article has been submitted, agree to be accountable for all aspects of the work criteria, made a significant contribution to the work reported, contributed to the article, and approved the submitted version.

## Conflict of Interest

The authors declare that the research was conducted in the absence of any commercial or financial relationships that could be construed as a potential conflict of interest.

## Publisher's Note

All claims expressed in this article are solely those of the authors and do not necessarily represent those of their affiliated organizations, or those of the publisher, the editors and the reviewers. Any product that may be evaluated in this article, or claim that may be made by its manufacturer, is not guaranteed or endorsed by the publisher.

## Abbreviations

AIC: Akaike information criterion
AJCC: American Joint Committee on Cancer
AUC: area under the curve
CSS: cancer-specific survival
DCC: distal cholangiocarcinoma
ELNN: examined lymph node number
LN: lymph node
LODDS: log odds of positive lymph nodes
LNR: lymph node ratio
PLNN: positive lymph node number
ROC: receiver operating characteristic
SEER, Surveillance: Epidemiology and End Results.
